# A Robust Effect Size Index

**DOI:** 10.1007/s11336-020-09698-2

**Published:** 2020-03-30

**Authors:** Simon Vandekar, Ran Tao, Jeffrey Blume

**Affiliations:** Department of Biostatistics, Vanderbilt University, 2525 West End Ave., #1136, Nashville, TN 37203 USA

**Keywords:** M-estimator, Cohen’s d, R square, standardized log odds, semiparametric

## Abstract

Effect size indices are useful tools in study design and reporting because they are unitless measures of association strength that do not depend on sample size. Existing effect size indices are developed for particular parametric models or population parameters. Here, we propose a robust effect size index based on M-estimators. This approach yields an index that is very generalizable because it is unitless across a wide range of models. We demonstrate that the new index is a function of Cohen’s *d*, $R^2$, and standardized log odds ratio when each of the parametric models is correctly specified. We show that existing effect size estimators are biased when the parametric models are incorrect (e.g., under unknown heteroskedasticity). We provide simple formulas to compute power and sample size and use simulations to assess the bias and standard error of the effect size estimator in finite samples. Because the new index is invariant across models, it has the potential to make communication and comprehension of effect size uniform across the behavioral sciences.

## Introduction

Effect sizes are unitless indices quantifying the association strength between dependent and independent variables. These indices are critical in study design when estimates of power are desired, but the exact scale of new measurement is unknown (Cohen, 1988), and in meta-analysis, where results are compiled across studies with measurements taken on different scales or outcomes modeled differently (Chinn, 2000; Morris and DeShon, 2002). With increasing skepticism of significance testing approaches (Trafimow and Earp, 2017; Wasserstein and Lazar, 2016; Harshman et al., 2016; Wasserstein et al., 2019), effect size indices are valuable in study reporting (Fritz et al., 2012) because they are minimally affected by sample size.

Effect sizes are also important in large open source datasets because inference procedures are not designed to estimate error rates of a single dataset that is used to address many different questions across tens to hundreds of studies. While effect sizes can have similar bias to *p*-values when choosing among multiple hypotheses, obtaining effect size estimates for parameters specified a priori may be more useful to guide future studies than hypothesis testing because, in large datasets, *p*-values can be small for clinically meaningless effect sizes.

There is extensive literature in the behavioral and psychological sciences describing effect size indices and conversion formulas between different indices (see e.g., Cohen, 1988; Borenstein et al., 2009; Hedges and Olkin, 1985; Ferguson, 2009; Rosenthal, 1994; Long and Freese, 2006). Cohen 1988 defined at least eight effect size indices for different models, different types of dependent and independent variables, and provided formulas to convert between the indices. For example, Cohen’s *d* is defined for mean differences, $R^2$ is used for simple linear regression, and standardized log odds ratio is used in logistic regression. Conversion formulas for some of these parametric indices are given in Table 1 and are widely used in research and software (Cohen, 1988; Borenstein et al., 2009; Lenhard and Lenhard, 2017).

Several authors have proposed robust effect size indices based on sample quantiles (Zhang and Schoeps, 1997; Hedges and Olkin, 1984). These are robust in the sense that they do not assume a particular probability model; however, they are defined as parameters in the sense that they are a specific functional of the underlying distribution.

**Table 1:** Effect size conversion formulas based on derivations from the robust index under homoskedasticity.

$\mapsto$	$|d |$	$f^2_\beta$	$R^2_\beta$	$S_\beta$
*d*	$|d |$	$(\pi _1^{-1} + \pi _0^{-1})^{-1}\times d^2$	$\frac{d^2}{(\pi _1^{-1} + \pi _0^{-1}) + d^2}$	$(\pi _1^{-1} + \pi _0^{-1})^{-1/2}\times |d |$
$f^2_\beta$	$(\pi _1^{-1} + \pi _0^{-1})^{1/2}\times \sqrt{f^2_\beta }$	$f^2_\beta$	$\frac{f_\beta ^2}{1+ f^2}$	$\sqrt{f^2_\beta }$
$R^2_\beta$	$(\pi _1^{-1} + \pi _0^{-1})^{1/2}\times \sqrt{\frac{R^2_\beta }{1-R^2}}$	$\frac{R^2_\beta }{1-R^2}$	$R^2_\beta$	$\sqrt{\frac{R^2_\beta }{1-R^2}}$
$S_\beta$	$(\pi _1^{-1} + \pi _0^{-1})^{1/2}\times S_\beta$	$S^2_\beta$	$\frac{S_\beta ^2}{1+ S^2}$	$S_\beta$

Each row denotes the input argument and the column denotes the desired output value. Robust versions of classical values can be obtained by computing them as a function of $S_\beta$. $\pi _1$ and $\pi _0$ denote the population proportions of each group for a two sample comparison. *d* is Cohen’s *d*, $f^2_\beta$ is Cohen’s effect size for multiple regression, $R^2_\beta$ is the partial coefficient of determination, $S_\beta$ is the robust index. The variables without subscripts denote the value for the full model including covariates. Conversion formulas derived by the robust index match classical formulas (Cohen, 1988; Borenstein et al., 2009; Lenhard and Lenhard, 2017)

Despite the array of effect sizes, there are several limitations to the available indices: 1) there is no single unifying theory that links effect size indices; 2) as defined, many effect size indices do not accommodate nuisance covariates or multivariate outcomes; and 3) each index is specific to a particular population parameter. For example, Cohen’s *d* is designed for mean differences in the absence of covariates, correlation is specific to linear regression, and existing semiparametric indices are quantile estimators. For these reasons, these classical effect size indices are not widely generalizable because their scale is dependent on the type of parameter.

In this paper, we define a new robust effect size index based on M-estimators. M-estimators are parameter estimators that can be defined as the maximizer of an estimating equation. This approach has several advantages over commonly used indices: a) The generality of M-estimators makes the index widely applicable across many types of models that satisfy mild regularity conditions, including mean and quantile estimators, so this framework serves as a canonical unifying theory to link common indices; b) the sandwich covariance estimate of M-estimators is consistent under model misspecification (MacKinnon and White, 1985; White, 1980), so the index can accommodate unknown complex relationships between second moments of multiple dependent variables and the independent variable; c) the robust effect size index is directly related to the Wald-style sandwich chi-squared statistic and is formulaically related to common indices.

Here, we describe sufficient conditions for the new effect size index to exist, describe how it relates to other indices, and show that other estimators can be biased under model misspecification. In three examples, we show that the new index can be written as a function of Cohen’s *d*, $R^2$, and standardized log odds ratio, demonstrating that it is related to indices that were developed using intuition for specific models. In addition, we describe how to obtain a simple estimate of the index and provide functions to compute power or sample size given an effect size index and degrees of freedom of the target parameter. Finally, we use simulations to assess the bias and standard error of the proposed index estimator. An R package to estimate the index is in development; the latest release is available at https://github.com/simonvandekar/RESI.

## Notation

Unless otherwise noted, capital letters denote vectors or scalars and boldface letters denote matrices; lower and upper case greek letters denote vector and matrix parameters, respectively. Let $W_1=\{Y_1, X_1\}, \ldots , W_n=\{Y_n, X_n\}$ be a sample of independent observations from $\mathbb {W} \subset \mathbb {R}^p$ with associated probability measure *G* and let *H* denote the conditional distribution of $Y_i$ given $X_i$. Here, $W_i$ denotes a combination of a potentially multivariate outcome vector $Y_i$ with a multivariate covariate vector $X_i$.

Let $W = \{W_1, \ldots , W_n\}$ denote the full dataset and $\theta ^* \mapsto \Psi (\theta ^*; W) \in \mathbb {R}$, $\theta ^* \in \mathbb {R}^{m}$ be an estimating equation,

1 $$\begin{aligned} \Psi (\theta ^*; W) = n^{-1}\sum _{i=1}^n \psi (\theta ^*; W_i), \end{aligned}$$

where $\psi$ is a known function. $\Psi$ is a scalar-valued function that can be maximized to obtain the M-estimator $\hat{\theta }$. We define the parameter $\theta$ as the maximizer of the expected value of the estimating equation $\Psi$ under the true distribution *G*,

2 $$\begin{aligned} \theta = \arg \max _{\theta ^* \in \Theta } \mathbb {E}_G \Psi (\theta ^* ; W) \end{aligned}$$

and the estimator $\hat{\theta }$ is

$$\begin{aligned} \hat{\theta } = \arg \max _{\theta ^* \in \Theta }\Psi (\theta ^* ; W). \end{aligned}$$

Assume,

3 $$\begin{aligned} \theta = (\alpha , \beta ), \end{aligned}$$

where $\alpha \in \mathbb {R}^{m_0}$ denotes a nuisance parameter, $\beta \in \mathbb {R}^{m_1}$ is the target parameter, and $m_0 + m_1 = m$.

We define the $m \times m$ matrices with *j*, *k*th elements

$$\begin{aligned} \begin{aligned} \mathbf {J}_{jk}(\theta )&= - \mathbb {E}_G\frac{\partial ^2 \Psi (\theta ^*; W)}{\partial \theta ^*_{j} \partial \theta ^*_{k} } \Big \vert _{\theta }\\ \mathbf {K}_{jk}(\theta )&= \mathbb {E}_G \frac{\partial \Psi (\theta ^*; W)}{\partial \theta ^*_{j} } \frac{\partial \Psi (\theta ^*; W)}{ \partial \theta ^*_{k} } \Big \vert _{\theta }, \end{aligned} \end{aligned}$$

which are components of the asymptotic robust covariance matrix of $\sqrt{n}(\hat{\theta } - \theta )$.

## A New Effect Size Index

### Definition

Here, we define a robust effect size that is based on the test statistic for

4 $$\begin{aligned} H_0: \beta =\beta _0. \end{aligned}$$

$\beta _0$ is a reference value used to define the index. Larger distances from $\beta _0$ represent larger effect sizes. Under the regularity conditions in the Appendix,

5 $$\begin{aligned} \sqrt{n}(\hat{\theta } - \theta ) \sim N\left\{ 0, \mathbf {J}(\theta )^{-1} \mathbf {K}(\theta ) \mathbf {J}(\theta )^{-1}\right\} . \end{aligned}$$

This implies that the typical robust Wald-style statistic for the test of (4) is approximately chi-squared on $m_1$ degrees of freedom,

6 $$\begin{aligned} T_{m_1}(\hat{\theta })^2 = n (\hat{\beta } - \beta _0)^T \Sigma _\beta (\hat{\theta })^{-1} (\hat{\beta }-\beta _0) \sim \chi ^2_{m_1}\left\{ n (\beta -\beta _0)^T \Sigma _\beta (\theta )^{-1} (\beta - \beta _0) \right\} , \end{aligned}$$

with noncentrality parameter $n (\beta -\beta _0)^T \Sigma _\beta (\theta )^{-1} (\beta - \beta _0)$, where $\Sigma _\beta (\theta )$ is the asymptotic covariance matrix of $\hat{\beta }$, and can be derived from the covariance of (5) (Boos and Stefanski, 2013; Van der Vaart, 2000). We define the square of the effect size index as the component of the chi-squared statistic that is due to the deviation of $\beta$ from the reference value:

7 $$\begin{aligned} S_\beta (\theta )^2 = (\beta -\beta _0)^T \Sigma _\beta (\theta )^{-1} (\beta - \beta _0). \end{aligned}$$

As we demonstrate in the examples below, the covariance $\Sigma _\beta (\theta )$ serves to standardize the parameter $\beta$ so that it is unitless. The regularity conditions given in Appendix are sufficient for the index to exist. The robust index, $S_\beta (\theta ) := \sqrt{S_\beta (\theta )^2}$, is defined as the square root of $S_\beta (\theta )^2$ so that the scale is proportional to that used for Cohen’s *d* (see Example 1).

This index has several advantages: it is widely applicable because it is constructed from M-estimators; it relies on a robust covariance estimate; it is directly related to the robust chi-squared statistic; it is related to classical indices, and induces several classical transformation formulas (Cohen, 1988; Borenstein et al., 2009; Lenhard and Lenhard, 2017).

### An Estimator

$S_\beta (\theta )$ is defined in terms of parameter values and so must be estimated from data when reported in a study. Let $T_{m_1}(\hat{\theta })^2$ be as defined in (6), then

8 $$\begin{aligned} \hat{S}_\beta (\theta ) = \left\{ \max \left[ 0, (T_{m_1}(\hat{\theta })^2 - m)/(n-m)\right] \right\} ^{1/2} \end{aligned}$$

is consistent for $S_\beta (\theta )$, which follows by the consistency of the components that make up $T_{m_1}(\hat{\theta })^2$ (Van der Vaart, 2000; White, 1980). We use the factor $(n-m)$ to account for the estimation of *m* parameters.

$\hat{S}_\beta (\theta )$ is the square root of an estimator for the noncentrality parameters of chisquared statistic. There is a small body of literature on this topic (Saxena and Alam, 1982; Chow, 1987; Neff and Strawderman, 1976; Kubokawa et al., 1993; Shao and Strawderman, 1995; López-Blázquez, 2000). While the estimator (8) is inadmissable (Chow, 1987), it has smaller risk than the usual unbiased maximum likelihood estimator (MLE), $S^2 = (T_{m_1}(\hat{\theta })^2 - m)/(n-m)$, because the MLE is not bounded by zero. We assess estimator bias in Sect. 7.

## Examples

In this section, we show that the robust index yields several classical effect size indices when the models are correctly specified. We demonstrate the interpretability of the effect size index through a series of examples. The following example shows that the robust index for a difference in means is proportional to Cohen’s *d*, provided that the parametric model is correctly specified; that is, the solution to the M-estimator is equal to the MLE.

### Example 1

(Difference in means) In this example, we consider a two mean model, where $W_i = \{Y_i, X_i\}$ and the conditional mean of $Y_i\in \mathbb {R}$ given $X_i$ converges. That is,

9 $$\begin{aligned} n_x^{-1} \sum _{i : X_i=x}^{n_x} \mathbb {E}( Y_{i} \mid X_i=x ) \xrightarrow {p} \mu _x \in \mathbb {R}, \end{aligned}$$

for independent observations $i=1,\ldots , n$, where $x,X_i \in \{0,1\}$, $n_x = \sum _{i=1}^n I(X_i=x)$, and we assume the limit (9) exists. In addition, we assume $\mathbb {P}(X_i =1)=\pi _1 = 1-\pi _0$ is known and that

$$\begin{aligned} n_x^{-1} \sum _{i : X_i=x}^{n_x}\text {Var}(Y_i \mid X_i=x) \xrightarrow {p} \sigma ^2_x < \infty . \end{aligned}$$

Let $$\partial \Psi (\theta ; W)/\partial \theta = n^{-1}\sum _{i=1}^n \{(2X_i - 1)\pi _{X_i}^{-1}Y_i - \theta \}$$, then

$$\begin{aligned} \hat{\theta }&= \frac{n_1}{n} \pi _1^{-1}\hat{\mu }_1 - \frac{n_0}{n} \pi _0^{-1}\hat{\mu }_0\\ \mathbb {E}\hat{\theta }&= \mu _1 - \mu _0 \\ J(\theta )&= 1 \\ K(\theta )&= \lim _{n\rightarrow \infty } n^{-1}\sum _{i,j} \mathbb {E}_H \left\{ (2 X_i - 1)\pi _{X_i}^{-1}Y_i - \theta \right\} \left\{ (2 X_j - 1) \pi _{X_j}^{-1}Y_j - \theta \right\} , \end{aligned}$$

where $\hat{\mu }_x = n_x^{-1} \sum _{i : X_i=x}^{n_x} Y_i$. When (9) holds, then $K(\theta ) = \lim _{n\rightarrow \infty } n^{-1}\sum _{i=1}^n \pi _{X_i}^{-2}\text {Var}(Y_i \mid X_i)$. Note that $\Psi$ in this example is not defined as the derivative of a log-likelihood: It defines a single parameter that is a difference in means and does not require each observation to have the same distribution. Despite this general approach, we are still able to determine the asymptotic variance of $n^{1/2}\hat{\theta }$,

$$\begin{aligned} J(\theta ) K(\theta )^{-1}J(\theta )&= \lim _{n\rightarrow \infty } n^{-1}\sum _{i=1}^n \pi _{X_i}^{-2}\text {Var}(Y_i \mid X_i) \\&= \lim _{n\rightarrow \infty } n^{-1}\left\{ n_1\pi _{1}^{-2}\sigma ^2_1 + n_0\pi _{0}^{-2}\sigma ^2_0 \right\} \\&= \pi _1^{-1}\sigma ^2_1 + \pi _0^{-1}\sigma ^2_0. \end{aligned}$$

Then the robust effect size (7) is

10 $$\begin{aligned} S_\beta (\theta ) = \sqrt{\frac{(\mu _1 - \mu _0)^2}{\pi _1^{-1}\sigma ^2_1 + \pi _0^{-1}\sigma ^2_0}}. \end{aligned}$$

For fixed sample proportions $\pi _0$ and $\pi _1$, when $\sigma _0^2 = \sigma _1^2$, $S_\beta (\theta )$ is proportional to the classical index of effect size for the comparison of two means, Cohen’s *d* (Cohen, 1988). However, $S_\beta (\theta )$ is more flexible: It can accommodate unequal variance among groups and accounts for the effect that unequal sample proportions has on the power of the test statistic. Thus, *S* is an index that accounts for all features of the study design that will affect the power to detect a difference. The robust index is proportional to the absolute value of the large sample z-statistic that does not rely on the equal variance assumption. This is what we expect in large samples when the equal variance assumption is not necessary for “correct” inference. In this example, we did not explicitly assume an identical distribution for all $X_i$, only that the mean of the variance of $Y_i$ given $X_i$ converges in probability to a constant.

The following example derives the robust effect size for simple linear regression. This is the continuous independent variable version of Cohen’s *d* and is related to $R^2$.

### Example 2

(Simple linear regression) Consider the simple linear regression model

$$\begin{aligned} Y_i = \alpha + X_i \beta + \epsilon _i \end{aligned}$$

where $\alpha$ and $\beta$ are unknown parameters, $Y_i \in \mathbb {R}$, $X_i \in \mathbb {R}$ and $\epsilon _i$ follows an unknown distribution with zero mean and conditional variance that can depend on $X_i$, $\text {Var}(Y_i \mid X_i) = \sigma ^2(X_i)$. Let $\Psi (\theta ; W_i) = n^{-1} \sum _{i=1}^n(Y_i - \alpha - X_i \beta )^2/2$. In this model

11 $$\begin{aligned} \begin{aligned} \mathbf {J}(\theta )^{-1}&= \sigma ^{-2}_x\begin{bmatrix} \sigma ^2_x + \mu ^2_x &{} - \mu _x \\ - \mu _x &{} 1 \\ \end{bmatrix} \\ \mathbf {K}(\theta )&= \begin{bmatrix} \sigma ^2 &{} \mu _{xy} \\ \mu _{xy} &{} \sigma ^2_{xy} +2 \mu _x \mu _{xy} - \mu _{x}^2\sigma ^2 \\ \end{bmatrix} \end{aligned} \end{aligned}$$

where

12 $$\begin{aligned} \begin{aligned} \mu _x&= \mathbb {E}_G X_i\\ \sigma ^2_x&= \mathbb {E}_G (X_i - \mu _x)^2\\ \sigma ^2&= \mathbb {E}_G (Y_i-\alpha -X_i\beta )^2\\ \mu _{xy}&= \mathbb {E}_G X_i(Y_i-\alpha -X_i\beta )^2\\ \sigma ^2_{xy}&= \mathbb {E}_G (X_i - \mu _x)^2(Y_i-\alpha -X_i\beta )^2. \end{aligned} \end{aligned}$$

After some algebra, combining the formulas (11) and (12) gives

$$\begin{aligned} \Sigma _\beta&= \sigma ^{-4}_x\sigma ^2_{xy}. \end{aligned}$$

Then (7) is

13 $$\begin{aligned} S_\beta (\theta )^2 = \frac{\sigma _x^4}{\sigma ^2_{xy}}\beta ^2. \end{aligned}$$

The intuition of (13) is best understood by considering the homoskedastic case where $\mathbb {E}_H (Y_i-\alpha -X_i\beta )^2 = \sigma ^2$ for all $i=1,\ldots , n$. Then, $\sigma _x^4/\sigma ^2_{xy} \beta ^2 = \sigma _x^2/\sigma ^2 \beta ^2$. This is similar to $R^2$, except that the denominator is the variance of $Y_i$ conditional on $X_i$ instead of the marginal variance of $Y_i$. The denominator of (13) accounts for the possible dependence between $X_i$ and $\text {Var}(Y_i \mid X_i)$.

In the following example, we introduce two levels of complexity by considering logistic regression with multidimensional nuisance and target parameters.

### Example 3

(Logistic regression with covariates) For logistic regression, we utilize the model

14 $$\begin{aligned} \mathbb {E}(Y_i \mid X_i) = \text {expit}(X_{i0} \alpha + X_{i1} \beta ) = \text {expit}(X_i \theta ), \end{aligned}$$

where $Y_i$ is a Bernoulli random variable, $X_i = [X_{i0}, X_{i1}] \in \mathbb {R}^{p-1}$ is a row vector, and $\alpha$ and $\beta$ are as defined in (3). Let $\mathbf {X} = [ X_1^T \ldots X_n^T]^T \in \mathbb {R}^{n \times (p-1)}$ and similarly define $\mathbf {X}_0$ and $\mathbf {X}_1$. Let $\mathbf {P} \in \mathbb {R}^{n \times n}$ be the matrix with $\mathbf {P}_{ii} = \text {expit}(X_i \theta )\left\{ 1-\text {expit}(X_i \theta )\right\}$ and $\mathbf {P}_{ij} = 0$ for $i\ne j$. Let $\mathbf {Q} \in \mathbb {R}^{n \times n}$ be the matrix with $\mathbf {Q}_{ii} = \{ Y_i - \text {expit}(X_i \theta )\}^2$ and $\mathbf {Q}_{ij} = 0$ for $i\ne j$. If (14) is correctly specified then $\mathbb {E}_{H}(\mathbf {P}_{ii} \mid X_i) = \mathbb {E}_{H}(\mathbf {Q}_{ii} \mid X_i) = \text {Var}(Y_i \mid X_i)$. If this equality does not hold, then there is under or over dispersion.

To find the robust effect size, we first need to find the covariance matrix of $\hat{\beta }$. To simplify notation, we define the matrices

$$\begin{aligned} \mathbf {A}_{k\ell }(\mathbf {P}) = \mathbb {E}_G n^{-1}\mathbf {X}_k^T \mathbf {P} \mathbf {X}_\ell \end{aligned}$$

for $k,\ell = 0,1$. The block matrix of $\mathbf {J}_G(\theta )^{-1}$ corresponding to the parameter $\beta$ is

15 $$\begin{aligned} \mathbf {I}_\beta (\theta )^{-1} = \left\{ \mathbf {A}_{11}(\mathbf {P}) - \mathbf {A}_{10}(\mathbf {P}) \mathbf {A}_{00}(\mathbf {P})^{-1} \mathbf {A}_{01}(\mathbf {P})\right\} ^{-1}. \end{aligned}$$

Equation (15) is the asymptotic covariance of $\hat{\beta }$, controlling for $\mathbf {X}_0$, if model (14) is correctly specified.

The robust covariance for $\beta$ can be derived by finding the block matrix of $J(\theta )^{-1} K(\theta ) J(\theta )^{-1}$ corresponding to $\beta$. In this general case, the asymptotic covariance matrix of $\hat{\beta }$ is

$$\begin{aligned} \Sigma _\beta (\theta ) =&\mathbf {I}_\beta (\theta )^{-1} \left[ \mathbf {A}_{10}(\mathbf {P}) \mathbf {A}_{00}(\mathbf {P})^{-1} \mathbf {A}_{00}(\mathbf {Q}) \mathbf {A}_{00}(\mathbf {P})^{-1} \mathbf {A}_{01}(\mathbf {P}) \right. \\&\left. - \mathbf {A}_{10}(\mathbf {P})\mathbf {A}_{00}(\mathbf {P})^{-1} \mathbf {A}_{01}(\mathbf {Q}) \right] \mathbf {I}_\beta (\theta )^{-1} \\&+ \mathbf {I}_\beta (\theta )^{-1} \left[ \mathbf {A}_{11}(\mathbf {Q}) - \mathbf {A}_{10}(\mathbf {Q})\mathbf {A}_{00}(\mathbf {P})^{-1} \mathbf {A}_{01}(\mathbf {P}) \right] \mathbf {I}_\beta (\theta )^{-1}. \end{aligned}$$

If the model is correctly specified, $\mathbf {P} = \mathbf {Q}$, $\Sigma _\beta (\theta ) = \mathbf {I}_\beta (\theta )^{-1}$, then

16 $$\begin{aligned} S_\beta (\theta ) = \sqrt{\beta ^T \mathbf {I}_\beta (\theta ) \beta }. \end{aligned}$$

The parameter (16) describes the effect of $\beta$ controlling for the collinearity of variables of interest $\mathbf {X}_1$, with the nuisance variables, $\mathbf {X}_0$. If the collinearity is high, then the diagonal of $\mathbf {I}_\beta (\theta )^{-1}$ will be large and the effect size will be reduced.

Many suggestions have been made to compute standardized coefficients in the context of logistic regression (for a review see Menard, 2004, 2011). When $m_1=1$, the square of the robust index in this context, under correct model specification, is the square of a fully standardized coefficient and differs by a factor of $\sqrt{n}$ from the earliest proposed standardized index (Goodman, 1972). The index proposed by Goodman (1972) is simply a Wald statistic and was rightly criticized for its dependence on the sample size (Menard, 2011), despite that it correctly accounts for the fact that the variance of a binomial random variable is functions of its mean, through the use of the diagonal matrix $\mathbf {P}$ in the matrix $\mathbf {I}_\beta (\theta )$. The robust index remediates the dependence that Goodman’s standardized coefficient has on the sample size.

## Relation to Other Indices

**Fig. 1:**
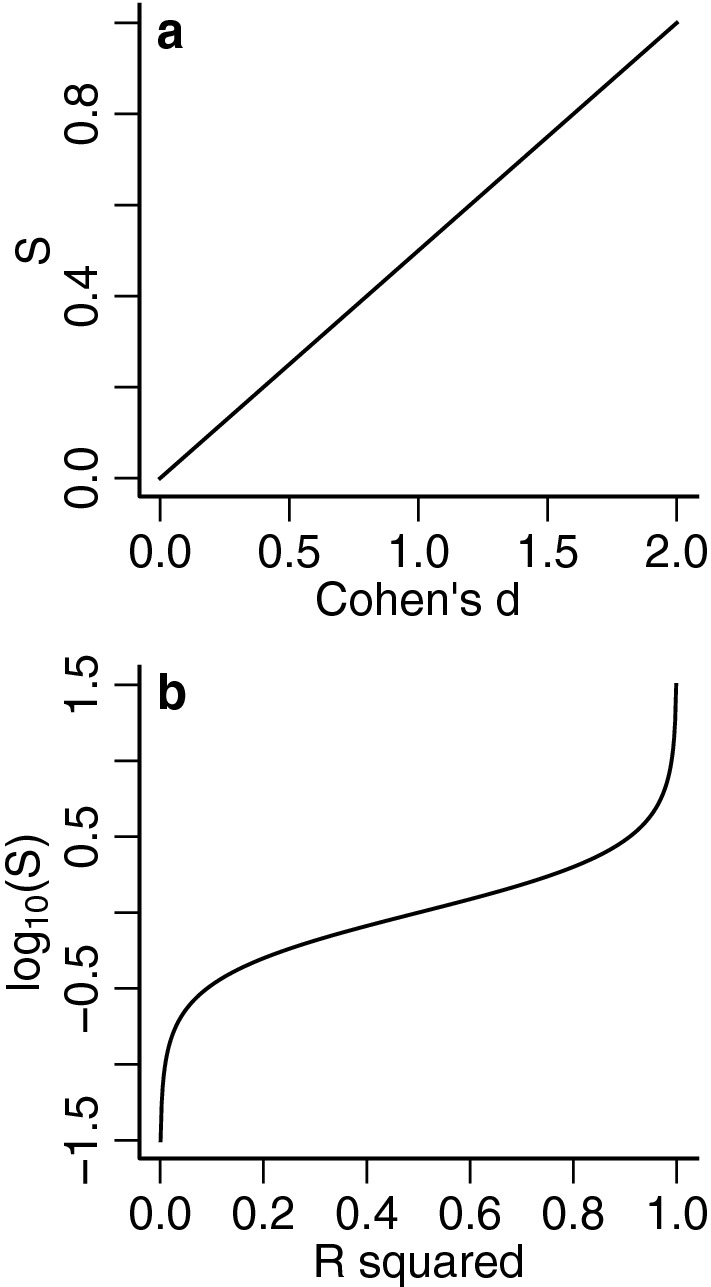
Graphs of the robust effect size as a function of some common effect size indices (see formulas in Table 1. **a** Cohen’s *d*, when $\pi _0=\pi _1=1/2$ and $\sigma _0 = \sigma _1$; **b** $R^2$.

The new index can be expressed as a function of several common effect size indices for continuous or dichotomous dependent variables when there is homoskedasticity (Fig. 1; Table 1). The relations between effect sizes implied by the new index are equivalent to the classical conversion formulas between effect sizes (Borenstein et al., 2009; Selya et al., 2012). While the index is related to existing indices under correct model specification, the advantage of the robust index is that it is defined if the variance model is incorrectly specified. This is the case, for example, in linear regression when there is heteroskedasticity and the model assumes a single variance term for all subjects or in logistic regression when there is overdispersion. By using the formulas in Table 1, we can obtain robust versions of classical indices by writing them as functions of $S_\beta$.

Cohen (1988) defined ranges of meaningful effect sizes for the behavioral sciences (Table 2). These intervals can also be used to define similar regions for the robust index. These recommendations serve as a useful guide, however, ranges of meaningful effect sizes are field specific and should be based on clinical expertise and the effect an intervention could have if applied to the population of interest.

**Table 2:** Effect size thresholds suggested by Cohen (1988) on the scale of *d* and the robust index ($S_\beta$), using the formula from Table 1 assuming equal sample proportions.

Effect size	*d*	*S*
None-small	[0, 0.2]	[0, 0.1]
Small-medium	(0.2, 0.5]	(0.1, 0.25]
Medium-large	(0.5, 0.8]	(0.25, 0.4]

### Bias of Existing Indices Under Model Misspecification

To understand the bias of the classical estimators under model misspecification, we compare the asymptotic value of the classical estimators to the effect size formulas in Table 1. Under model misspecification, the existing parametric effect size indices can be biased.

The estimator for Cohen’s *d* using pooled variance converges to

$$\begin{aligned} \hat{d}_C = \frac{\hat{\mu }_1 - \hat{\mu }_0}{\frac{(n_1-1)\hat{\sigma }_1^2 + (n_0-1)\hat{\sigma }_0^2}{n_1 + n_0 -2} } \xrightarrow {p} \frac{\mu _1-\mu _0}{\pi _1 \sigma ^2_1 + (1-\pi _1)\sigma ^2_0} = d_C. \end{aligned}$$

Taking the ratio of this value to the robust value of Cohen’s *d* in Table 1 gives

$$\begin{aligned} d_C/d(S) = (\pi _1^{-1} + (1-\pi _1)^{-1})^{-1/2} \times \left( \frac{\pi _1^{-1} \sigma ^2_1 + (1-\pi _1)^{-1} \sigma ^2_0}{\pi _1 \sigma ^2_1 + (1-\pi _1) \sigma ^2_0}\right) ^{1/2} \end{aligned}$$

**Fig. 2:**
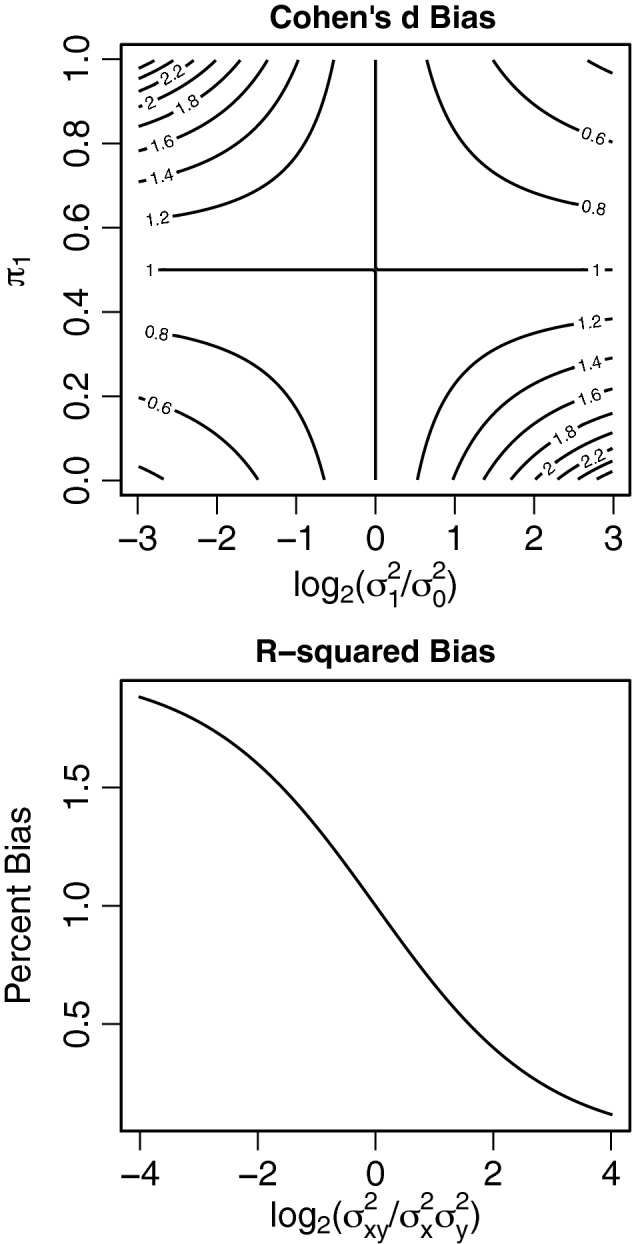
Percent bias for Cohen’s *d* and $R^2$. When $\pi _1=1/2$ or the variances are equal the classical estimator of Cohen’s *d* is unbiased, it can be positively or negatively biased when the variances and sampling proportions are not equal. Similarly for $R^2$, when $\text {Var}(Y_i \mid X_i)$ is constant across subjects, there is no bias (because $\sigma ^2_{xy}=\sigma ^2_x \sigma ^2_y$), but when this is not true, the classical estimator can be positively or negatively biased depending on the relationship between the variances. Variables are as defined in (12).

A plot of this ratio with respect to $\log _2(\sigma ^2_1/\sigma ^2_0)$ and $\pi _1$ is given in Fig. 2. When $\pi _1 = 1/2$ or $\sigma ^2_1 = \sigma ^2_0$ then there is no bias. When $\pi _1<1/2$ and $\sigma ^2_1>\sigma ^2_0$ Cohen’s *d* overestimates the effect size. When $\pi _1<1/2$ is small and $\sigma ^2_1<\sigma ^2_0$ Cohen’s *d* under underestimates the effect size. The plot is symmetric about the point (0, 1/2).

The classical estimator for $R^2$ converges to

$$\begin{aligned} R^2_C = \frac{\sigma ^2_x \beta ^2}{\sigma ^2_x \beta ^2 + \sigma ^2_y}. \end{aligned}$$

Taking the ratio of this value and the formula for $R^2(S)$ given in Table 1 gives,

$$\begin{aligned} R^2_C/R^2(S_\beta ) = \frac{\sigma ^4_x \beta ^2 + \sigma ^2_x\sigma ^2_y}{\sigma ^4_x \beta ^2 + \sigma ^2_{xy}}, \end{aligned}$$

where variables are as defined in (12). Figure 2 plots the bias as a function of $\log _2\{\sigma ^2_{xy}/(\sigma ^2_x\sigma ^2_y)\}$. When the variance is constant across subjects, $\mathrm {Var}(Y_i \mid X_i) = \sigma ^2_y$, then the bias is zero. If not, then the direction of the bias of the classical estimator depends on the relationship between $\mathrm {Var}(Y_i \mid X_i)$ and $X_i$.

## Determining Effect Sizes, Sample Sizes, and Power

**Fig. 3:**
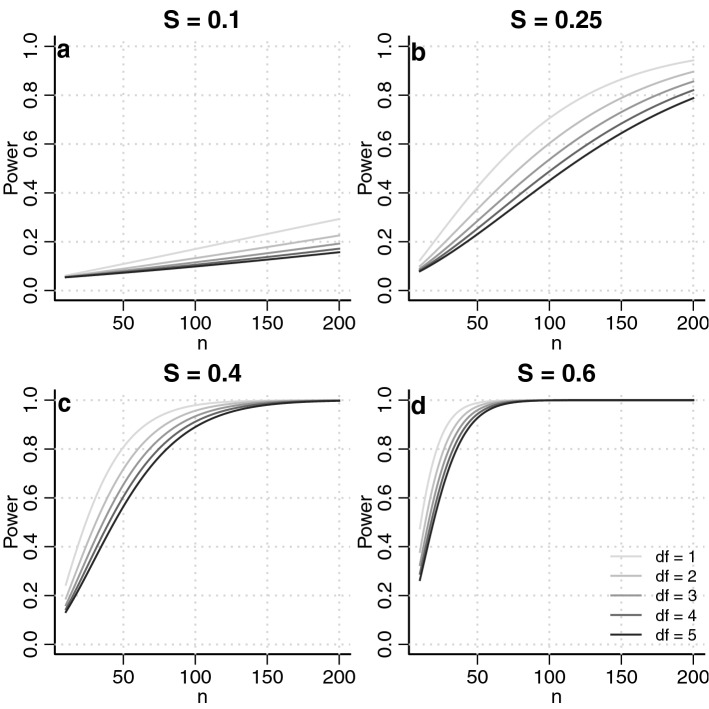
Power curves as a function of the sample size for several values of the robust index (*S*) and degrees of freedom (df), for a rejection threshold of $\alpha =0.05$. The curves are given by formula (17) and are not model dependent.

A convenient aspect of the robust index is that it makes asymptotic power calculations easy. The formula is the same for every parameter that is a solution to an estimating equation, such as (2). For a fixed sample size and rejection threshold, power can be determined from the robust index and degrees of freedom of the chi-squared test using (6). The explicit formula for power can be written

17 $$\begin{aligned} 1- t_2 = 1-\Phi _\text {df}\left\{ \Phi ^{-1}_\text {df}(1- t_1; 0); n\times S_\beta (\theta )^2 \right\} , \end{aligned}$$

where $t_1$ and $t_2$ denote the type 1 and type 2 error rates, respectively, df denotes the degrees of freedom of the test statistic, $\Phi (\cdot ; \lambda )$ denotes the cumulative distribution function of a noncentral chi-squared distribution with noncentrality parameter $\lambda$, and $S_\beta$ is as defined in (7). Equation (17) can be easily solved for sample size, power, error rate, or effect size, using basic statistical software with fixed values of the other variables (Fig. 3). Because the robust index is not model dependent, power curves are effectively model-free and applicable for any fixed sample size, rejection threshold, and degrees of freedom.

**Fig. 4:**
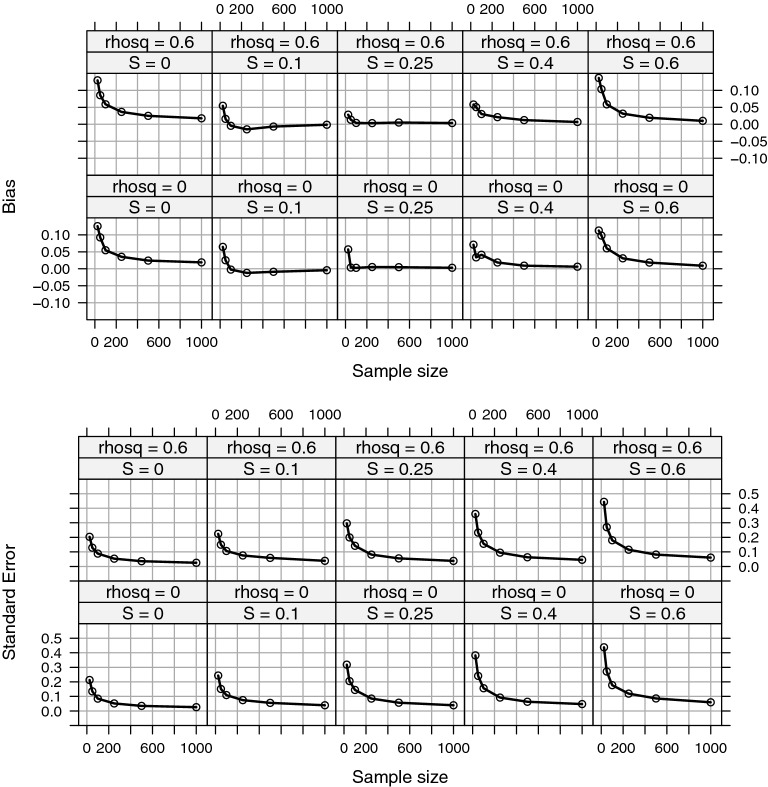
Bias and standard error of $\hat{S}$ when the data generating distribution has skew$=$0.63 with two nuisance covariates ($m_0=2$). $\hat{S}$ tends to be positively biased across values of *S*. The standard error is proportional to *S* and is quite large in small samples. Rhosq denotes the total squared correlation of nuisance covariates with the target variables. Rhosq does not affect the bias, standard error, or value of the effect size index because *S* is defined conditionally on the covariates.

## Simulation Analysis

We used 1,000 simulations to assess finite sample bias and standard errors of the estimator (8). Covariates of row vectors, $X_i$, were generated from a multivariate normal distribution $X_i \sim N(0, \Sigma _X)$, where,

$$\begin{aligned} \Sigma _X= \begin{bmatrix} \mathbf {I}_{m_0} &{} \rho ^2/(m_0 m_1) \times \varvec{1}_{m_0}\varvec{1}_{m_1}^T\\ \rho ^2/(m_0 m_1) \times \varvec{1}_{m_1}\varvec{1}_{m_0}^T&{} \mathbf {I}_{m_1} \end{bmatrix} \end{aligned}$$

with $\rho ^2 \in \{0, 0.6\}$, $m_0 \in \{2, 5\}$, and $m_1 \in \{1,3,5\}$. Here, $\mathbf {I}_{m_0}$ and $\varvec{1}_{m_0}$ denote the $m_0\times m_0$ identity matrix and a vector of ones in $\mathbb {R}^{m_0}$, respectively. This distribution implies that the total correlation between the nuisance covariates and target covariates is equal to $\rho ^2$. Samples of $Y_i$, for $i=1, \ldots , n$ of size $n \in \{25, 50, 100, 250, 500, 1000\}$ were generated with mean

$$\begin{aligned} \mathbb {E}Y_i = \beta X_{i1}\varvec{1}_{m_1}, \end{aligned}$$

where $\beta$ was determined such that $S \in \{0, 0.1, 0.25, 0.4, 0.6 \}$. We used a gamma distribution with shape parameter $a\in \{0.5, 10 \}$ and rate equal to $\sqrt{a/ X^2_{i,m_0+1}}$ to generate heteroskedastic errors for $Y_i$. For each simulation, we compute bias of the estimator (8). Only a subset of the results are reported here; however, code to run the simulations and the saved simulation results are published with this paper.

Bias and standard error of the estimator is presented for $\rho ^2\in \{0, 0.6\}$, for $m_0=2$ , and all values of *S* considered in the simulations (Fig. 4). Results demonstrate the effect size estimator is biased upwards in small samples, but the bias is close to zero for sample sizes over 500. Because the effect size is defined conditional on covariates, the existence of covariates does not affect estimation bias. The standard error of the estimator is larger in small samples and for larger values of *S*. When the sample size is small, $n=25$, the standard error can be larger than the value of $S_\beta$.

## Discussion

We proposed a robust effect size index that utilizes an M-estimator framework to define an index that is generalizable across a wide range of models. The robust index provides a unifying framework for formulaically relating effect sizes across different models. The proposed index is robust to model misspecification, has an easily computable estimator, and is related to classical effect size indices. We showed that classical estimators can be asymptotically biased when the covariance model is misspecified.

The relationship between the robust index and indices based on correctly specified models (such as Cohen’s *d* and $R^2$) is appealing because it follows intuition from other areas of robust covariance estimation. That is, when the estimating equation is proportional to the log-likelihood, then the robust index is a function of classical definitions derived from likelihood-based models. The new framework also generalizes classical indices by easily accommodating nuisance covariates and sandwich covariance estimators that are robust to heteroskedasticity. The robust index puts effect sizes for all models on the same scale so that asymptotically accurate power analyses can be performed for model parameters using a single framework.

One important feature of the proposed index is that it is defined conditional on covariates. While the effect size lies on a standardized scale that is related directly to the power of the test, the inclusion of covariates affects the interpretation of the index because it is defined conditional on the covariates. For this reason, careful consideration of the target parameter is necessary for accurate interpretation and comparison across studies that present the robust index. Marginal estimators (without conditioning on covariates) should be considered if the investigator is interested in the general effect across a given population.

Several limitations may inspire future research topics: Like p-values, estimates of effect size indices can be subject to bias by data dredging. Also, the estimator can be biased in small samples because the index is based on asymptotic results. Thus, methods for bias adjustment or low mean squared error estimators could be considered to adjust the effects of data dredging or small sample sizes. Here, we considered an M-estimator framework, but a semiparametric or robust likelihood framework may have useful properties as well (Royall and Tsou, 2003; Blume et al., 2007). We believe this index serves as a first step in constructing a class of general robust effect size estimators that can make communication of effect sizes uniform across models in the behavioral sciences.

## Appendix

The following regularity conditions are required for the asymptotic normality of $\sqrt{n}(\hat{\theta } - \theta )$ (Van der Vaart, 2000)

- The function $\theta ^* \mapsto \Psi (\theta ^*; w)$ is almost surely differentiable at $\theta$ with respect to *G*, where objects are as defined in (1) and (2).
- For every $\theta _1$ and $\theta _2$ in a neighborhood of $\theta$ and measurable function *m*(*w*) such that $\mathbb {E}_G m(W)^2 < \infty$, $|\Psi (\theta _1;w) - \Psi (\theta _2;w) |\le m(w) \Vert \theta _1 - \theta _2\Vert$.
- The function $\theta ^* \mapsto \mathbb {E}_G \Psi (\theta ^*; W)$ admits a second-order Taylor expansion at $\theta$ with a non-singular second derivative matrix $\mathbf {J}(\theta )$.
- $$\Psi (\hat{\theta },W) \ge \sup _{\theta ^*}\Psi (\theta ^*,W) - o_p(n^{-1})$$ and $$\hat{\theta } \xrightarrow {p} \theta $$.
